# Impact of chronic rhinosinusitis on severe asthma patients

**DOI:** 10.1371/journal.pone.0171047

**Published:** 2017-02-15

**Authors:** Ta-Jen Lee, Chia-Hsiang Fu, Chun-Hua Wang, Chi-Che Huang, Chien-Chia Huang, Po-Hung Chang, Yi-Wei Chen, Chia-Chen Wu, Ching-Lung Wu, Han-Pin Kuo

**Affiliations:** 1 Division of Rhinology, Department of Otolaryngology, Chang Gung Memorial Hospital and Chang Gung University, Taoyuan, Taiwan; 2 Graduate Institute of Clinical Medical Sciences, College of Medicine, Chang Gung University, Taoyuan, Taiwan; 3 Department of Thoracic Medicine, Chang Gung Memorial Hospital, Taipei, Taiwan; National and Kapodistrian University of Athens, GREECE

## Abstract

Coexistence of chronic rhinosinusitis (CRS) with asthma appears to impair asthma control. Type-2 innate lymphoid cells (ILC2s) respond to the cytokines of thymic stromal lymphopoietin (TSLP), interleukin (IL)-25 and IL-33, thus contributing to airway diseases such as CRS and asthma. We investigate whether the augmented Th_2_-cytokines in CRS might be related to sinonasal tract ILC2s corresponding to enhanced IL-25, IL-33 and TSLP release in severe asthmatics, and be involved in asthma control. Twenty-eight asthmatics (12 non-severe and 16 severe) with CRS receiving nasal surgery were enrolled. The predicted FEV_1_ inversely associated with CRS severity of CT or endoscopy scores. Higher expression of Th2-driven cytokines (IL-4, IL-5, IL-9, and IL-13), TSLP, IL-25 and IL-33 in nasal tissues was observed in severe asthma. Severe asthmatics had higher ILC2 cell counts in their nasal tissues. ILC2 counts were positively correlated with Th_2_-cytokines. Nasal surgery significantly improved asthma control and lung function decline in severe asthma and CRS. The higher expression of IL-33/ILC2 axis-directed type 2 immune responses in nasal tissue of CRS brought the greater decline of lung function in severe asthma. ILC2-induced the upregulated activity of Th_2_-related cytokines in asthmatics with CRS may contribute to a recalcitrant status of asthma control.

## Introduction

Asthma and chronic rhinosinusitis (CRS), seemingly two distinct diseases, are instead viewed as similar common airway diseases under the consideration of a united airway concept [1,2]. There is a strong association between CRS and asthma. The extent of chronic rhinosinusitis had a direct relationship to low airway inflammation in patients with severe asthma, and nearly half of those patients had undergone previous surgery for sinusitis [3,4]. The coexistence of CRS with asthma appears to impair asthma control [3,5]. Further, an advanced sinus disease has been documented to be a powerful, independent risk factor for diffcult asthma [6,7]. The long-term asthma control, including symptom scores, steroid dependence and emergency department visit, would improve and remain stable after functional endoscopic sinus surgery (FESS) treatment for asthmatic patients with CRS [8].

The link between CRS and asthma, two diseases in different location of airways, would be complex. A type 2 helper T (Th2)-driven cytokine pattern has been proposed as the potential major drivers for the development of unstable asthma, and improvement of CRS in asthmatics can reverse the cytokine profiles [9]. Increased levels of interleukin (IL)-4 and IL-13 were observed in the sinus lavage of CRS patients, and greater amounts of IL-13 mRNA-positive cells were found in the sinus epithelium of CRS patients [10,11]. Anti-IL-4 treatment has been reported effective in reducing nasal polyp burden in CRS patients with nasal polyps [12]. Levels of IL-3, IL-4, and IL-5 correlated well with the eosinophil counts in tissue in asthma with CRS [13]. These cytokines may impact epithelial and smooth muscle cells of lower airway, contributing to airway hyper-responsiveness (AHR), mucus hypersecretion and subepithelial fibrosis [14]. IL-13 also induces production of several matrix metalloproteinases, resulting in airway remodeling [15].

The adaptive Th_2_ cells are no longer thought to be the only source of Th_2_-related cytokines. Epithelial cell-derived cytokines, including thymic stromal lymphopoietin (TSLP), IL-25, and IL-33 are critical regulators of innate and adaptive immune responses associated with Th_2_ cytokine-mediated inflammation in asthma [16]. These cytokines are upstream of IL-4 and IL-13 and may have greater therapeutic potential, since they play an important “gate keeper” role in mucosal homeostasis [17]. The type-2 innate lymphoid cells (ILC2s) respond to the cytokines IL-25, IL-33 and TSLP, and produce the effector cytokines IL-4, IL-5, IL-9, IL-13 and amphiregulin [18,19]. Since ILC2s have been detected in airway tissues [20], thus we hypothesize that the augmented Th_2_-cytokines in nasal tissue from CRS patients with asthma may be derived from sinonasal tract ILC2s in response to enhanced IL-25, IL-33 and TSLP release. Thus, an increase in Th_2_-cytokines expression in nasal tissues in CRS patients with severe asthma might be associated with a greater number of ILC2, when compared with CRS patients with non-severe asthma.

## Materials and methods

### Study populations

Twenty-eight asthmatic patients who met the diagnostic criteria of Global Initiative for Asthma (GINA) guidelines [21] were prospectively transferred from outpatient clinics of Thoracic Medicine Department in Chang Gung Memorial Hospital for rhinologic examination from March to December 2014. Sixteen patients with severe asthma (aged 57.6 ± 3.3 years, 6 women and 10 men) required either continuous or near-continuous oral corticosteroids, high-dose inhaled corticosteroids, or both to achieve a level of mild-to-moderate persistent asthma [22]. Twelve patients with non-severe asthma (aged 58.6 ± 3.8 years, 2 women and 10 men) used inhaled beclomethasone (0–1000 μg/d or equivalent) with perfect control of their asthma symptoms. Presence of CRS was defined by the criteria of European Position Paper on Rhinosinusitis and Nasal Polyps (EPOS) [23]. All of these patients with CRS and asthma received functional endoscopic sinus surgery (FESS) after 3-month maximal medical treatment, including nasal tropic corticosteroids and broad spectrum oral antibiotics, to control their chronic sinusitis. Patients with histories of previous nasal surgery, recent upper airway infection or systemic corticosteroid usage within 4 weeks were excluded from this study. This study was approved by the Ethics Committee of Chang Gung Memorial Hospital (No. 101-5069B). The study was conducted in accordance with the principles of Helsinki Declaration. A written informed consent was obtained from all the subjects.

Basic demographic data were recorded, including age, gender, pulmonary function tests, sinus disease severity (Lund-Kennedy endoscopy score and Lund-Mackay computed tomography (CT) score, and smoking status. The pulmonary function tests, including forced vital capacity (FVC), forced expiratory volume in 1 second (FEV_1_) and FEV_1_/FVC ratio were measured the day before nose surgery. The yearly decline in FEV_1_ or FVC was measured by the change in FEV_1_ within 5 years prior to the study. Allergy status, blood tests, questionnaires for subjective disease severity, including Sino-Nasal Outcome Test-22 (SNOT-22) for rhinologic symptoms and asthma control test (ACT) score were measured before nasal surgery. Unscheduled clinic or emergency department visit for exacerbation of asthma was recorded as well. Nasal endoscopy, pulmonary function tests and ACT scores were performed 3 months after nasal surgery.

### Nasal tissue preparation, immunostaining and confocal laser microscopic analysis

Mucosa tissue specimens of the middle turbinate facing middle meatus were obtained for patients with non-severe and severe asthma with CRS. Nasal mucosal tissue specimens (3×4 mm) were rinsed in phosphate buffered saline (PBS) pH 7.6, and then processed for polymerase chain reaction (PCR). Nasal tissue specimens were fixed in 10% formalin and embedded in paraffin. After being de-waxed thoroughly in xylene and rinsed in absolute alcohol, sections are incubated in 3% H_2_O_2_ for 30 minutes to quench endogenous peroxidase. Then the sections were microwaved in citric acid buffer with 0.1% Triton for 5 minutes to enhance antigen exposure followed by incubation in a 0.2% normal swine serum (DAKO, CA, USA) for 30 min to block the positive and negative charges of tissues. Afterwards, the sections were subjected to an one hour incubation with the specific IL-4, IL-5, IL-9, IL-13, IL-25, IL-33, and TSLP antibodies (diluted 1:100) or the nonspecific purified rabbit IgG (diluted 1:100) as a control. Antibody labeling was visualized using an avidin-biotin complex method (LSAB 2 kit; DAKO, CA, and DAB peroxidase substrate kit; Vector Laboratories, Burlingame, CA). Nasal tissues were spun down on slide then fixed in methanol at -20°C for 5 min. The cells were then blocked with 1% BSA/PBS at room temperature for 30 min and incubated with the primary mouse anti-human monoclonal antibodies, followed by anti-mouse. Secondary antibodies were used for the detection of ST2R, CD25, or CRTH2 (Molecular Probes, Eugene, Ore) with FITC, PE and Cy3 conjugates (Sigma-Aldrich, Stockholm, Sweden) to detect the expression of ILC2 cells. Nuclear staining was performed. After washing and air drying, the cells were mounted with anti-fade mounting medium (Dako Cytomation). Images were acquired with a confocal laser-scanning microscope (Leica) and analyzed by Metamorph Image Analysis (Universal Imaging). Positively stained cells in merged images were counted to compare the number ILC2s in each group, and presented as the cell counts in each mm^3^.

### Real-time PCR analysis

The mRNA expression of IL-4, IL-5, IL-9, IL-13, IL-25, IL-33, and TSLP in nasal mucosa was analyzed by real time PCR. Total cellular RNA was extracted from nasal mucosa and purified using an RNeasy mini kit (Qiagen GmbH, Strasse, Germany). cDNAs were prepared from 1 μg of total RNA via reverse transcription at 37°C for 60 minutes and at 95°C for 5 minutes using the High capacity RNA-to-cDNA kit (Applied Biosystems, Foster City, CA, USA) according to the manufacturers' protocols. Quantitative real-time PCR was carried out using the TaqMan assay with primers specific for target genes (see S1 Table) and Applied Biosystems 7500 Fast Real Time PCR System (Applied Biosystems). The quantitative real-time PCR assay was based on primers that specifically amplify our target genes. The primers and fluorogenic probes for RNA mentioned above and GADPH were purchased from Applied Biosystems. The amplification efficiency of the specific primers and GADPH are validated in a preliminary experiment. For the PCR analysis, each sample was run in triplicates in separate tubes to permit quantification of the genes of target cytokines normalized to GADPH. The PCR condition consisted of initial denaturation step of 95°C for 20 seconds, followed by 50 cycles of amplification at 95°C for 3 seconds and at 60°C for 30 seconds. Data analysis was performed using 7500 software version 2.0.4 (Applied Biosystems). The level of expression of target mRNA was determined as the ΔCT method according to the manufacturer's instructions (Applied Biosystems: Relative quantization of gene expression ABI Prism 7700 sequence detection system. The threshold cycles were used to calculate arbitrary mRNA concentrations by the difference of Ct values between samples and calibrator (qPCR Human Reference Total RNA, Clontech Laboratories). The presentation of cytokines mRNA was normalized to the level of GADPH mRNA.

### Statistical analysis

The data expressed as mean ± standard error of mean (SEM). Statistical significance for intergroup comparisons was determined by Mann-Whitney U test for continuous variables and Fisher’s exact test for categorical variables. The correlations between demographic data, clinical laboratory tests, expression of cytokines in nasal tissue, as well as clinical symptom scores, endoscopy scores, CT scores, pulmonary function tests, ACT scores, cytokine levels, and ILC2 cell counts were determined using the Spearman's coefficient. All statistical analysis was performed using the GraphPad Prism (version 5) software package (GraphPad Prism Software, Inc, San Diego, CA). *P* value less than 0.05 is significant.

## Results

### Clinical characteristics of study populations

**Fig 1:**
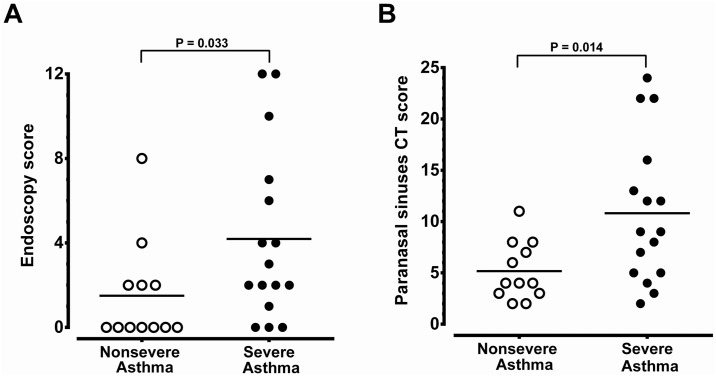
Comparison of the severity of CRS between non-severe asthma group (n = 12) and severe asthma group (n = 16). The results based on the comparison of endoscopy score (A) and paranasal sinus CT score (B). The significance is indicated.

**Fig 2:**
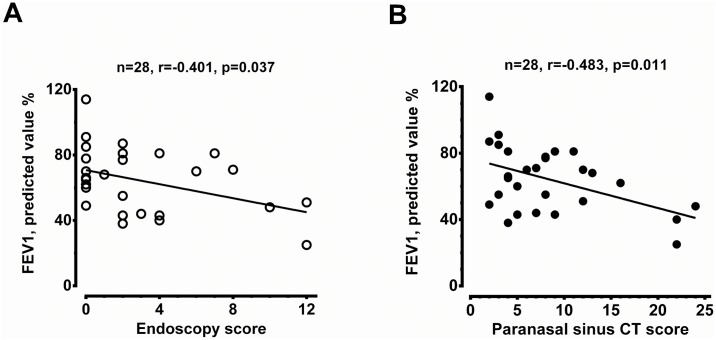
Correlation between predicted FEV_1_ value (%) and CRS severity. The results were presented in endoscopy score (A) and paranasal sinus CT score (B) for all asthmatic patients. The patient number and significance are indicated.

**Fig 3:**
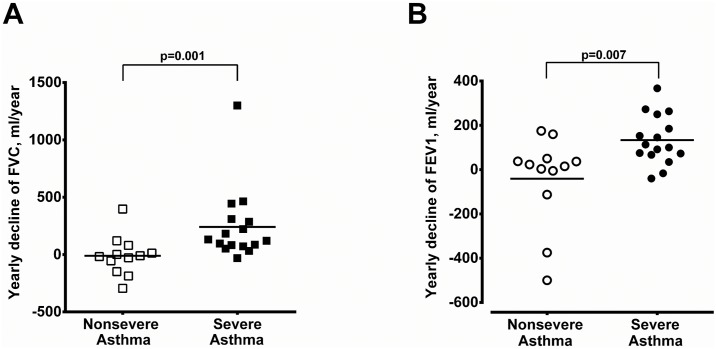
Yearly decline of pulmonary function before nasal surgery. Yearly decline of FVC (A) and FEV_1_ (B) before nasal surgery in non-severe and severe asthmatics with CRS.

The demographic data of 28 asthmatic patients were summarized in Table 1. The non-severe asthmatics had better pulmonary tests and ACT score. Patients with severe asthma and CRS received more combination therapy, anti-IgE therapy and oral corticosteroids in the control of asthma compared to those of non-severe asthmatics with CRS. There was no difference between these two groups, in terms of age, gender, atopy, blood IgE levels, coexistence with CRS or nasal symptom-specific SNOT-22 scores. The severe asthmatics tended to have higher peripheral eosinophil counts although the difference did not reached a significant level (*P* = 0.114). The lack of difference in eosinophil counts may be attributed to a greater number of severe asthmatics under prednisolone treatment (Table 1). Patients with severe asthma had significantly higher CRS severity in terms of endoscopy scores and paranasal sinus CT scores when compared to non-severe asthma patients (*P* = 0.033 and 0.034, respectively) (Fig 1A and 1B). A significant negative correlation was observed between pre-operative FEV_1_ predicted value and endoscopy scores or paranasal sinus CT scores (Fig 2A and 2B). Patient with severe asthma and CRS had a greater yearly decline in either FVC or FEV_1_ compared to patients with non-severe asthma and CRS (Fig 3A and 3B, respectively).

**Table 1:** Clinical characteristics of study subjects.

Characteristic	Non-severe Asthma	Severe Asthma
** Number**	**12**	**16**
** Age (y)**	**58.6 ± 3.8**	**57.6 ± 3.3**
** Gender: female/male**	**2/10**	**6/10**
** FVC predicted value (%)**	**82.8 ± 4.9**	**64.8 ± 4.4**†
** FEV**_**1**_ **predicted value (%)**	**78.1 ± 4.3**	**50.1 ± 4.3**†
** FEV**_**1**_**/FVC (%)**	**87.5 ± 3.0**	**73.8 ± 3.4**†
** ACT score**	**24.1 ± 0.3**	**19.7 ± 0.6**†
** Current smoker (n)**	**6**	**5**
** Atopy (n)**	**5**	**8**
** Serum IgE (IU/ml)**	**588.6 ± 275.6**	**410.2 ± 126.0**
** Eosinophil count (%)**	**1.9 ± 0.8**	**5.1 ± 1.3**
** SNOT-22 score**	**64.6 ± 8.6**	**51.1 ± 8.0**
**Medication over previous year**^1^		
** ICS alone**	**3**	**0**
** ICS + LABA**	**8**	**12**
** ICS + LABA + LAMA**	**1**	**4**
** Daily oral corticosteroids**^2^	**0**	**5**

Data expressed as mean ± SEM. FVC, forced vital capacity; FEV_1_, forced expiratory volume in 1 second; ACT, asthma control test; IgE, immunoglobulin E; SNOT-22, Sino-nasal outcome test-22; ICS, inhaled corticosteroids; LABA, long-acting beta-2 agonist; LAMA, Long-acting muscarinic antagonist.

^†^P < 0.01 compared with non-severe asthma.

^1^P < 0.01 analyzed by Chi-square test for trend.

^2^Daily dose of prednisolone was 5 to 10 mg/day.

### Expression of TSLP, IL-25, IL-33, IL-4, IL-5, IL-9 and IL-13 in nasal tissues

**Fig 4:**
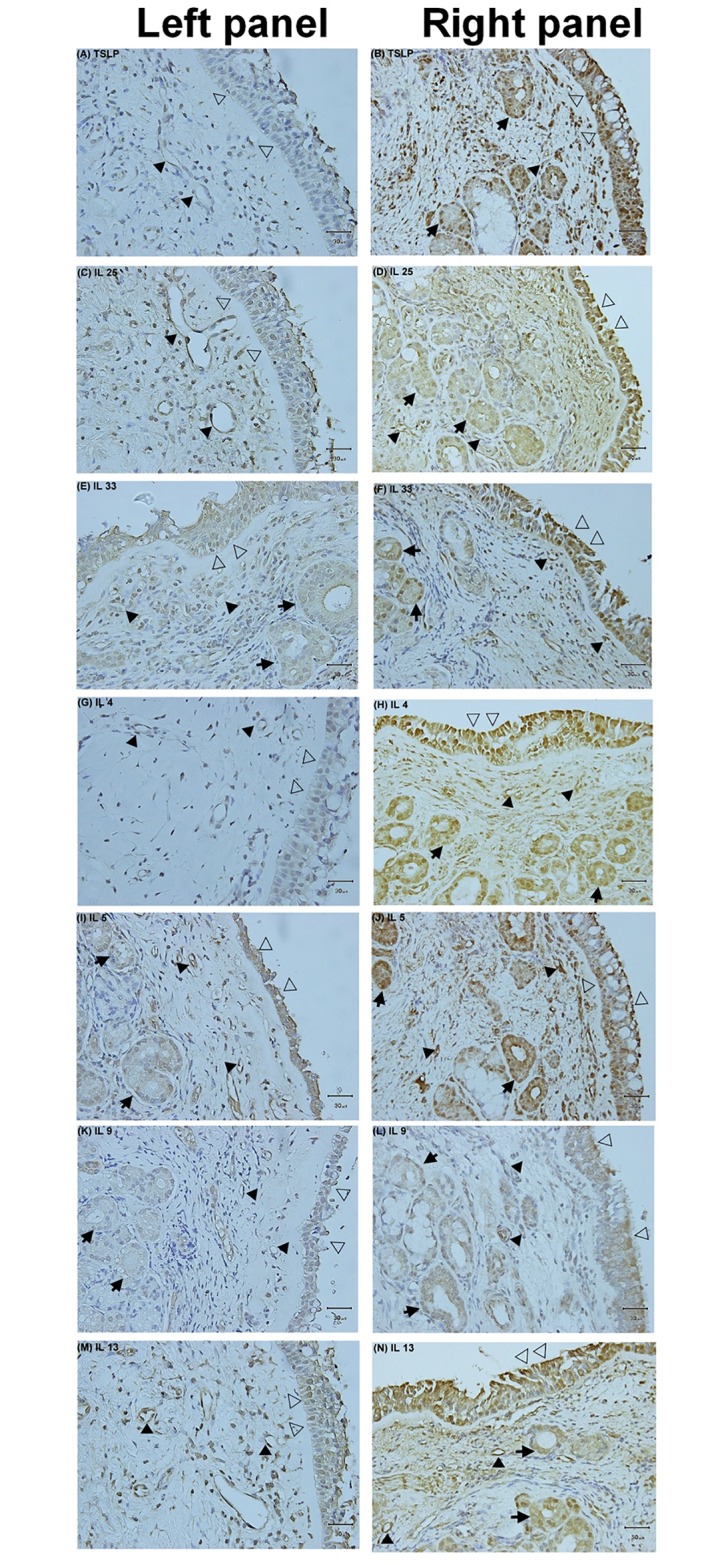
Immunohistochemistry studies in nasal mucosa. The expression of TSLP, IL-25, IL-33, IL-4, IL-5, IL-9 and IL-13 in nasal tissues derived from patients with non-severe asthma (left panel, A, C, E, F, G, I, K and M) or severe asthma (right panel, B, D, F, H, J, L and N) with CRS. The magnification is 400X. Open arrow = epithelium, close arrow = endothelium, arrow = mucus gland.

There was a strong expression of TSLP, IL-25, and IL-33 in the epithelium, the endothelium of small vessels and subepithelial infiltrating cells in the nasal tissue derived from severe asthma patients with CRS (Fig 4B, 4D and 4F, respectively). In contrast, the expression of TSLP, IL-25, and IL-33 in non-severe asthma patients with CRS group was very weak (Fig 4A, 4C and 4E, respectively).

**Fig 5:**
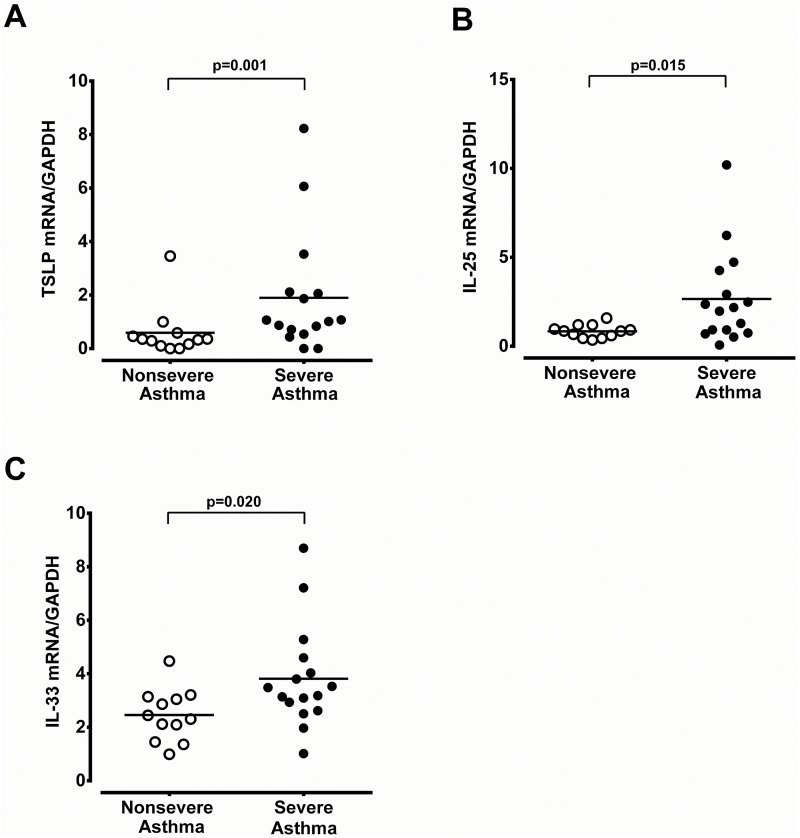
The mRNA expression of TSLP, IL-25 and IL-33 in nasal tissue. Real-time PCR analysis of TSLP (A), IL-25 (B), and IL-33 (C) mRNA expression in nasal tissues derived from non-severe and severe asthmatics with CRS. The significance is indicated.

**Fig 6:**
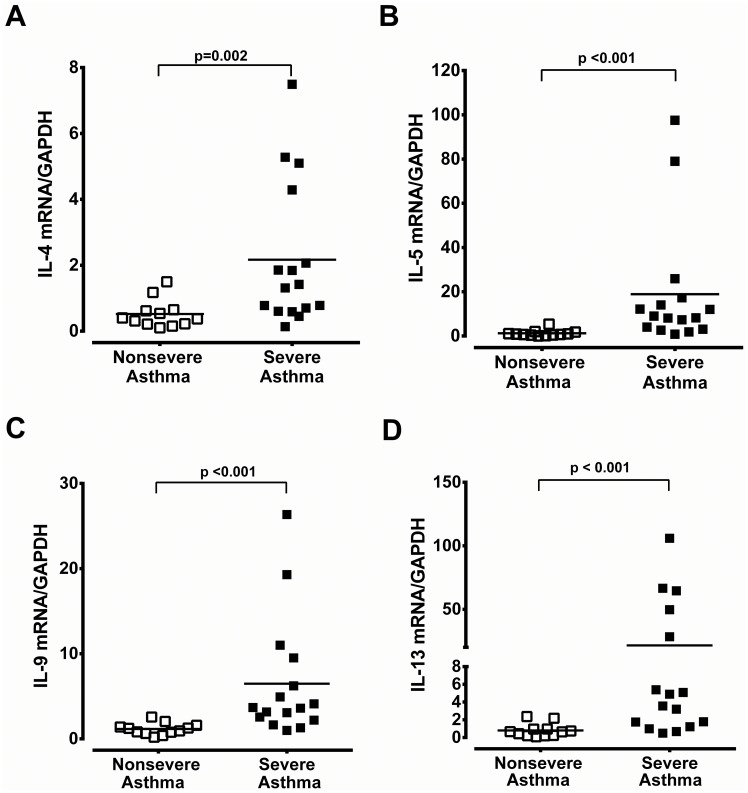
The mRNA expression of Th2- cytokines in nasal tissue. Real-time PCR analysis of IL-4 (A), IL-5 (B), IL-9 (C), and IL-13 (D) mRNA expression in nasal tissue derived from patients with non-severe and severe asthma with CRS. The significance is indicated.

Immunoreactivity of Th_2_-derived cytokines (IL-4, IL-5 and IL-13) was only weakly detected in the epithelium of nasal biopsy specimens of non-severe asthma patients with CRS (Fig 4G, 4I and 4M, respectively). Conversely, in severe asthma with CRS, Th_2_-derived cytokines of IL-4, IL-5, and IL-13 were markedly expressed in the epithelium, endothelium, subepithelial infiltrating cells, and mucus glands (Fig 4F, 4J and 4M, respectively). IL-9 immunoreactivity was found to a less magnitude in the epithelium, endothelium, and mucus glands in patients with severe asthma with CRS (Fig 4L). The mRNA expression of TSLP, IL-25 and IL-33 was significantly higher in patients with severe asthma and CRS compared to those of non-severe asthma with CRS (Fig 5A–5C). The Th_2_-derived cytokines, IL-4, IL-5, IL-9, and IL-13 mRNA expression was also markedly increased in the patients with severe asthma patients with CRS (Fig 6A–6D).

### ILC2 cells in nasal tissues

**Fig 7:**
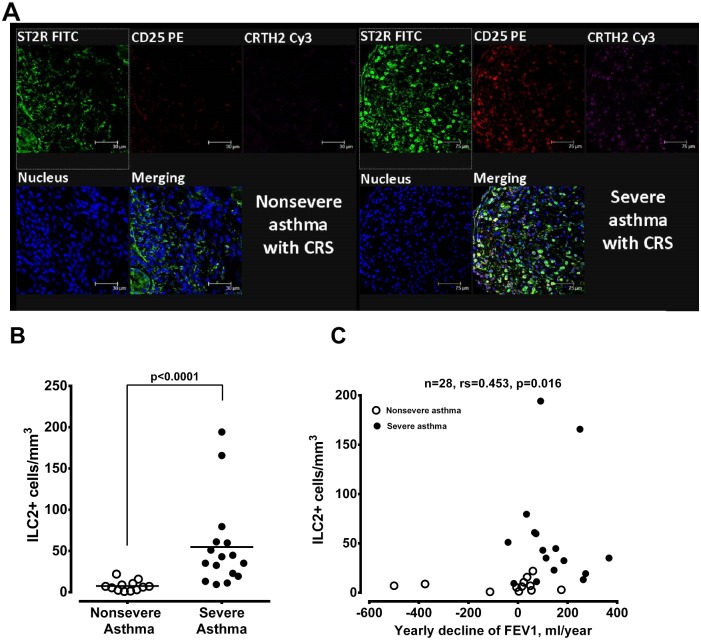
The presentation of ILC2 in nasal tissue and its relation with yearly decline of pulmonary function. (A) Simultaneous immunocytochemical staining of nasal tissues derived from non-severe (left panel) and severe asthmatics (right panel) for ST2 receptor (green), CD25 (red) and CRTH2 (purple) to represent ILC2+ cells that were positive for triple immunostaining (ST2R^+^/CD25^+^/CRTH2^+^) cells (white). (B) The mean cell number of immunostained ILC2+ cells per mm^3^ in nasal tissues derived from non-severe and severe asthmatics. The significance is indicated. (C) Correlation of the ILC2+ cell numbers in nasal tissues and yearly decline in FEV_1_ of non-severe and severe asthma. The patient number and significance are indicated.

To investigate whether the ILC2+ cells, a key player to bridge the innate immunity, were involved in the up-regulation of the corresponding Th_2_-derived cytokines in nasal tissues, ILC2+ cells were identified by immunostaining with ST2R (IL-33 receptor), CRTH2 (receptor of prostaglandin D_2_) and CD25 (α-chain of IL-2 receptor) in nasal mucosa tissues. Increased number of ILC2 cells was detected in nasal tissue derived from patients with severe asthma and CRS, while rare in non-severe asthmatics with CRS (Fig 7A and 7B, respectively). There was a significant correlation between cell number of ILC2+ cell and the levels of IL-13, IL-5 or IL-9 mRNA expression in nasal tissues (Table 2). The yearly decline in FEV_1_ was significantly correlated with the cell numbers of ILC2+ cells in the nasal tissues derived from of patients with severe and non-severe asthma with CRS (Fig 7C).

**Table 2:** Correlation between number of ILC2+ cells and Th_2_ cytokine mRNA in nasal tissue.

Cytokines	R	N	P value*
**IL-13**	**0.582**	**28**	**0.001**
**IL-4**	**0.299**	**28**	**0.123**
**IL-5**	**0.661**	**28**	**< 0.001**
**IL-9**	**0.577**	**28**	**0.001**

*The analysis was made by Spearman’s test.

### Pulmonary function and asthma control before and after nasal surgery

**Fig 8:**
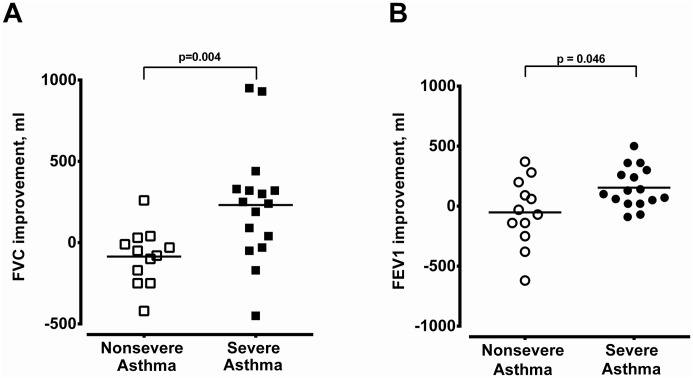
The improvement in pulmonary function after operation. The improvements in FVC (A) and FEV_1_ (B) of non-severe and severe asthmatics with CRS 3 months after nasal surgery.

Sixteen severe asthma patients with CRS and 12 non-severe asthma patients with CRS received nasal surgery. Severe asthma patients with CRS had better improvements in FVC and FEV_1_ 3 months after nasal surgery compared with non-severe asthma patients (Fig 8A and 8B). Furthermore, asthma control in terms of ACT score was significantly improved from 19.7 ± 0.6 to 23.4 ± 0.3 (n = 16, *P* < 0.001) in severe asthma patients 3 months after surgery, while the ACT score was not changed in non-severe asthma patients (from 24.1 ± 0.3 to 24.1 ± 0.2, n = 12).

### The effect of nasal polyps

Five asthmatics patients with CRS had nasal polyps in this investigation, including one in non-severe asthma group and the other four in severe asthma group. Patients with nasal polyps had significant greater endoscopy score and CT score (both *P* = 0.011). Besides, in severe asthma group, compared with CRS patients without nasal polyps, those with nasal polyps had higher expression of IL-13 (*P* = 0.004) and IL-5 (*P* = 0.009) in nasal tissue but similar results in other cytokines, ILC2 cell numbers, yearly decline in pulmonary functions, and post-operative ACT scores. If we excluded all patients with nasal polyps, severe asthmatics (n = 12) still had significantly higher expression in IL-33-axis cytokines, Th_2_ cytokines, ILC2 cells, yearly decline of pulmonary functions, and post-operative improvements in ACT scores than those in non-severe asthmatics (n = 11).

## Discussion

Coexistence with CRS has been well known to hinder asthma control. Medical treatment of CRS improves asthma control and reduces AHR [24]. Although nasal blockage or aspiration of nasal contents have been proposed as possible contributing factors, a systemic response seems to play a more important role in this relationship [25]. This study has provided pro-inflammatory cytokine profiles in nasal tissues of CRS linking with severe asthma and asthma control. Our results have demonstrated that the severity of CRS is associated with asthma stability and negatively correlated with the pulmonary function. Pre-operative subjective quality of life scores do not always correlate with the objective measurements for severity of sinus diseases judged by endoscopy scores or CT scores [26,27]. Thus, severe asthmatics with CRS in this investigation had higher endoscopy scores and CT scores but similar SNOT-22 scores. Nevertheless, surgical treatment of CRS significantly improved asthma control and pulmonary function, which further confirmed the impact of CRS on asthma control.

We have demonstrated an increased expression of Th_2_-cytokines, IL-4, IL-5, IL-9, and IL-13 at either protein or mRNA levels in nasal tissues of severe asthmatics compared with non-severe asthmatics. Increased gene expression or secretion of the Th_2_-cytokines has been established in poor control of asthma [28,29]. These Th_2_-cytokines promote the survival and migration of eosinophils, enhance airway hyper-responsiveness [30], increase mucus production and transformation of airway fibroblasts to myofibroblasts, leading to airway remodeling and airflow limitation in asthma [30]. A blockage of IL-4Rα or IL-13, thus inhibiting IL-4 and/or IL-13 activity in asthma, has been shown in preventing asthma exacerbations in the context of withdrawal of LABA and ICS, increases in FEV_1_ and reductions in the usage of short-acting beta-2 agonists in patients with moderate to severe asthma [31,32]. After removal of the nasal polyposis or nasal tissue, the pulmonary function of FEV_1_ and FVC and symptom score were significantly improved in severe asthma patients but not in non-severe asthma, suggesting the Th_2_-derived cytokines from CRS nasal tissues may be implicated in the pathogenesis of severe asthma.

Although the cellular source of Th_2_-derived cytokines is not elucidated in this study, it may be partly related to the release of IL-25, IL-33 and TSLP. IL-33, IL-25 and TSLP released from mucosal surface have been shown to activate type 2 innate lymphoid cells (ILC2), which directly secrete Th2 cytokines [18,19], and dendritic cells leading to the production of IgE via B cells [33]. IL-33 also directly acts on CD4^+^ T cells, mast cells and eosinophils to aggravate adaptive phase of type 2 immune responses [34]. In animal studies, IL-33 and to a less extent, TSLP are involved in mediating both innate and adaptive chronic type 2 immune responses to chronic exposure to natural airborne allergens [35], including airway inflammation and IgE antibody production. In severe asthma patients with CRS, there was an upregulated expression of IL-33, IL-25 and TSLP in their nasal tissues, compared to those in non-severe asthma patients. There was also an increased number of ILC2 in CRS nasal tissues of severe asthma patients. The cell number of ILC2 in CRS nasal tissues was significantly correlated with mRNA expression of Th_2_-cytokines, IL-5, IL-9, and IL-13. Our results are compatible with previous reports that functional importance of ILC2 is involved in Th_2_-mediated inflammatory process of chronic rhinosinusitis [36]. ILC2s-directed Th_2_ cytokines in nasal tissues may directly or aggravate adaptive Th_2_ immunity to augment lower airway inflammation, leading to the subsequent unstable status of severe asthma.

ILC2 are lineage negative lymphocytes, that is, they are negative for surface expression of known lineage markers. In humans, the combinations of CD1a, CD3, CD4, CD11b, CD11c, CD14, CD16, CD19, CD20, CD123, TCRb, TCRd, CD235a, and FceR1 have been used to exclude analogous lineage-positive cells [37,38]. Positive markers for human ILC2 include CRTH2 (prostaglandin D2 receptors), IL-33R, IL-7R, T1/ST2, CD161, c-kit [37]. In this study, we did not exclude analogous lineage-positive cells, but the co-existence of ST2 and CRTH2 seems to be specific for IL-25, IL-33 responsive ILC2 cells [37].

All subjects in this study received nasal corticosteroids and anti-histamine for at least 3 months. The persistent existence of Th_2_-cytokines in CRS nasal tissues of patients with severe asthma indicates that the release of these cytokines is corticosteroid insensitive. It is still controversial whether ILC2 cells are corticosteroid resistant [39,40]. Corticosteroids have been shown to induce ILC2 cells apoptosis in one report [39]. On the other hand, TSLP has been shown to confer ILC2 cell partial resistance to corticosteroids. Thus, it is unknown in this study whether the high number of ILC2 cells in CRS nasal tissues of severe asthma is corticosteroid resistant and persistently releases Th_2_-cytokines with nasal corticosteroid therapy. Since severe asthma is associated with corticosteroid resistance, it is also possible that the persistent existence of Th_2_-cytokines in CRS nasal tissues may be attributed to systemic corticosteroid resistance of severe asthma. The supposed role of ILC2 cells is drawn based on observational correlations, and further investigation with more patients, longer duration of follow-up would be requisite to conclude a more definite causative mechanism.

In summary, patients with severe asthma had more severe CRS endoscopically and radiologically than patients with non-severe asthma. An enriched presentation of IL-33/ILC2 axis-directed Th_2_ cytokines in CRS nasal tissue leads to a rapid decline in lung function associated with poor asthma control. Future novel therapeutics targeting IL-33/ILC2-associated type 2 immunity might be beneficial to severe asthmatics with CRS who are refractory to current standard medical treatments.

## Supporting information

S1 Table Primer sequences used for RT-PCR. Forward and reverse primers for real-time PCR were listed.
(DOC)
