# Supplementary material for: Impact of chronic rhinosinusitis on severe asthma patients
Source: PLoS One. 2017 Feb 15;12(2):e0171047. doi: 10.1371/journal.pone.0171047 (PMC5310870; doi:10.1371/journal.pone.0171047)
Supplement: S1 Table — Forward and reverse primers for real-time PCR were listed. (DOC) [file pone.0171047.s001.doc]

**Impact of Chronic Rhinosinusitis on Severe Asthma Patients**

Ta-Jen Lee^1,*^, Chia-Hsiang Fu^1,2,*^, Chun-Hua Wang^3^, Chi-Che Huang^1,2^, Chien-Chia Huang^1,2^, Po-Hung Chang^1,2^, Yi-Wei Chen^1^, Chia-Chen Wu^1^, Ching-Lung Wu^1^, Han-Pin Kuo^3^

**S1 Table.** **Primer sequences used for RT-PCR.**

|  | **Forward primers** | **Reverse primers** |
| --- | --- | --- |
| **IL-4** | 5’-TTTGCTGCCTCCAAGAACACA-3’ | 5’-TCCTGTCGAGCCGTTTCAG-3’ |
| **IL-5** | 5’-AGACCTTGGCACTGCTTTCT-3’ | 5’-CAGTACCCCCTTGCACAGTT-3’ |
| **IL-9** | 5’-TGCAGTGCTAATGTGACCAGT-3’ | 5’-TCTCACTGAAGCATGGCTTGG-3’ |
| **IL-13** | 5’-GAGCTGGTCAACATCACCCA-3’ | 5’-AGCTGTCAGGTTGATGCTCC-3’ |
| **IL-25** | 5’-CTGGAGGCTGGTCCCTTTTT-3’ | 5’-CTGCTCCAGACAGCACTTCA-3’ |
| **IL-33** | 5’-GCTCTCTGAAACTTAGTTGATGG-3’ | 5’-CCTTAGATGTCACCTGTCTCTT-3’ |
| **TSLP** | 5’-CCGAGTTCAACAACACCGTC-3’ | 5’-GGTGGGATTGAAGGTTAGGCT-3’ |
| **GADPH** | 5’-TTCCAGGAGCGAGATCCCT-3’ | 5’-CACCCATGACGAACATGGG-3’ |
